# Prediction of tuberculosis clusters in the riverine municipalities of the Brazilian Amazon with machine learning

**DOI:** 10.1590/1980-549720240024

**Published:** 2024-05-13

**Authors:** Luis Silva, Luise Gomes da Motta, Lynn Eberly

**Affiliations:** I University of Minnesota, Minneapolis – Minneapolis (MN), United States.; II Universidade Federal Fluminense – Niterói (RJ), Brazil.

**Keywords:** Tuberculosis, Amazon, Spatial analysis, Machine learning, Epidemiology, Ribeirinhos, Tuberculose, Amazônia, Análise espacial, Aprendizado de máquina, Epidemiologia, Ribeirinhos

## Abstract

**Objective:**

Tuberculosis (TB) is the second most deadly infectious disease globally, posing a significant burden in Brazil and its Amazonian region. This study focused on the “riverine municipalities” and hypothesizes the presence of TB clusters in the area. We also aimed to train a machine learning model to differentiate municipalities classified as hot spots vs. non-hot spots using disease surveillance variables as predictors.

**Methods:**

Data regarding the incidence of TB from 2019 to 2022 in the riverine town was collected from the Brazilian Health Ministry Informatics Department. Moran’s I was used to assess global spatial autocorrelation, while the Getis-Ord GI* method was employed to detect high and low-incidence clusters. A Random Forest machine-learning model was trained using surveillance variables related to TB cases to predict hot spots among non-hot spot municipalities.

**Results:**

Our analysis revealed distinct geographical clusters with high and low TB incidence following a west-to-east distribution pattern. The Random Forest Classification model utilizes six surveillance variables to predict hot vs. non-hot spots. The machine learning model achieved an Area Under the Receiver Operator Curve (AUC-ROC) of 0.81.

**Conclusion:**

Municipalities with higher percentages of recurrent cases, deaths due to TB, antibiotic regimen changes, percentage of new cases, and cases with smoking history were the best predictors of hot spots. This prediction method can be leveraged to identify the municipalities at the highest risk of being hot spots for the disease, aiding policymakers with an evidenced-based tool to direct resource allocation for disease control in the riverine municipalities.

## RESUMO

**Objetivo:**

A tuberculose (TB) é a segunda doença infecciosa que mais mata no mundo, representando um problema de saúde pública no Brasil, especialmente na região amazônica. Este estudo analisa a TB nos municípios ribeirinhos” com o objetivo de identificar aglomerados de alta incidência, também conhecidos como “*hot spots*”. Posteriormente, utilizando aprendizagem de máquina, visamos prever estes aglomerados por meio de variáveis de vigilância epidemiológica. Assim buscamos auxiliar o ente público no combate à TB nesta região.

**Métodos:**

Dados da incidência de TB nos “municípios ribeirinhos” foram coletados entre os anos de 2019 e 2022 do Departamento de Informática do Ministério da Saúde. O índice de Moran foi utilizado para a determinação de autocorrelação espacial global, enquanto o método Getis-Ord GI* foi empregado para a autocorrelação espacial local. Variáveis referentes ao diagnóstico, tratamento e características socioeconômicas associadas aos casos foram utilizadas para a predição de aglomerados de alta incidência por meio de um modelo Random Forest.

**Resultados:**

Foram identificados aglomerados com alta incidência de TB a oeste e baixa incidência a leste. O total de seis variáveis de vigilância epidemiológica foi identificado como relevante para a predição. Nosso modelo *Random Forest* alcança uma área sob a curva da característica operacional do receptor (AUC-ROC) de 0,81.

**Conclusão:**

Municípios com altas porcentagens de casos recorrentes, mortes por TB, mudança do esquema de tratamento, casos novos e casos com história de tabagismo estão associados a aglomerados de alta incidência. Esperamos que este método de identificação de possíveis aglomerados de TB seja útil para o ente público no combate à doença na região.

## INTRODUCTION

A 2022 report by the World Health Organization places tuberculosis (TB) as the second most deadly infectious disease globally, surpassed only recently by COVID-19^1^. It also shows Brazil as one of the 30 countries with the highest TB burden in the world. Brazilian healthcare authorities have reported 78,057 cases of the disease in 2022. As such, the yearly incidence of TB in the country was 34.9 per 100 thousand^2^.

Among Brazilian states above the national average of TB incidence, many are in the country’s Amazonian Region. In fact, of all states in the so-called Legal Amazon, only Rondônia is below the national annual incidence average^2^. Legal Amazon is a lawfully defined territory, encompassing all municipalities in the country where the Amazonian Biome is predominant^3^. Although relevant for public administration concerns, this definition fails to consider the diversity of geographical and socioeconomic characteristics of the Brazilian Amazon.

More specifically, the municipalities that the main rivers of the Amazon basin pass through share important social determinants of health and should be studied as a separate epidemiological population. Due to its poor-quality soil^4^, economic structure based on agroforestry^5^ and reliance on rivers as the primary mean of transportation^6^, municipalities in the Legal Amazon that are intersected by an “economically viable waterway,” as designated by the federal authority National Water Agency^7^ , are here defined as riverine municipalities.

Our study hypothesizes that TB incidence in these municipalities exhibits global and local spatial autocorrelation. It also aims to train a machine-learning (ML) model that uses surveillance variables to predict municipalities classified as high-incidence clusters, known as hot spots, among municipalities classified as non-hot spots. These variables are related to TB care in each municipality and include socioeconomic, diagnostic, and treatment characteristics of cases. Healthcare professionals are responsible for actively collecting this information when diagnosing a case of TB in any municipality in Brazil. They must file a report to the federal authorities using a standard chart containing information about a case’s medical history, current TB characteristics, and relevant complementary exams performed for the specific care of TB. Each case is later compiled into a national health informatics surveillance system, and data is made publicly available by the Health Ministry of Brazil.

The overall goal is to develop an epidemiological tool that can predict municipalities with a high likelihood of being hot spots and identify the most important surveillance variables related to this task. As such, we hope to aid this understudied region by providing a data-driven approach to assist in resource allocation for the control of TB.

## METHODS

Primary data were extracted from the Sistema de Informação de Agravos de Notificação — SINAN (National Information System for Disease Notification). The Brazilian Health Ministry Informatics Department (DATASUS) makes the data for this surveillance system publicly available through its portal TABNET, which is a federal repository for healthcare data related to the country’s Universal Healthcare System. Area-level data for TB cases in municipalities classified as riverine were collected from 2019 to 2022 and merged into a single dataset. More specifically, the variable utilized to determine inclusion in the study was municipality of residence, guaranteeing that each TB case corresponds to the area of interest.

Surveillance variables associated with each case represent the percentage of TB cases in that municipality with specific socioeconomic, disease or healthcare delivery characteristics. Supplementary material 1 has a complete list of all variables considered in this analysis and a brief explanation of their meaning.

Given that data is at the municipality level and that it is publicly available, the Institutional Review Board of the University of Minnesota deemed this investigation as “human subjects exempt” (Supplementary material 2).

Global spatial cluster analysis for the overall cumulative incidence of TB in the riverine municipalities from 2019 to 2022 was performed using the Global Moran’s I method for area-level data, employing the queen adjacency approach to determine neighbors^8^. The significance of the clustering was estimated by Monte Carlo simulation (n=1,000,000). Local determination of hot spots was conducted through the Optimized Getis Ord-Gi*^9^.

To enhance ML model performance, the Boruta method^10^ was used to select surveillance variables more likely to be relevant for predicting hot spots. This method determines variable importance through the creation of new random variables by shuffling cell values between rows and comparing their performance against original variables in the dataset. Comparison is made by performing multiple Random Forest classification models, and, in each iteration, different variables are removed, and model accuracy is evaluated. Variables with a better mean accuracy than the randomly generated ones are considered relevant for further analysis^11^. Variables were selected for inclusion in the prediction model if they were superior to the best randomly generated variables; the comparison was made based on the median Z-score for accuracy.

After variable selection, a Random Forest classification model was trained to predict hot spots among non-hot spots in the riverine municipalities. Random Forest classification is a ML approach that uses sample bootstrapping and weak learning aggregation from decision trees to create a model that can predict predetermined classes of data points (i.e., supervised learning)^12^.

The advantage of using Random Forest is that aggregation is achieved from an assembly of decision trees. When using decision trees for classification problems, the dataset is split at a cut-off point for a random variable in an attempt to perfectly separate classes; in our case, to separate hot spots from non-hot spots. The model’s cut-off points from each variable can be inferred from data visualization.

Random Forest models have been shown to be among the best-performing ML models for multiple tasks in healthcare, including both clinical^13-15^ and public health prediction problems^16-18^. Moreover, it has been compared with other ML models in spatial cluster prediction and has emerged as the superior method for this task^19^.

The dataset was split into training and testing sets in a 70:30 ratio, with previous work having demonstrated the consistent advantages of this training split strategy in healthcare data regardless of prediction model chosen^20^. Model performance evaluation was done through a “cross-validation k-fold” strategy with k = 10. This cross-validation method has been associated with a reliable accuracy performance estimation when compared to similar ML evaluation strategies^21,22^, and was deemed adequate for the current analysis. Hyperparameter tuning in our training dataset yielded a Random Forest classification model with the number of randomly drawn candidate variables of 1 and 5 thousand trees in each aggregation.

Supplementary material 3 is a visual representation of the methods utilized in this study to answer our main research question.

Determination of local spatial clusters was performed with the software ArcMap version 10.8.2, while all other analysis were made using R version 4.2.0.

## RESULTS

**Figure 1:**
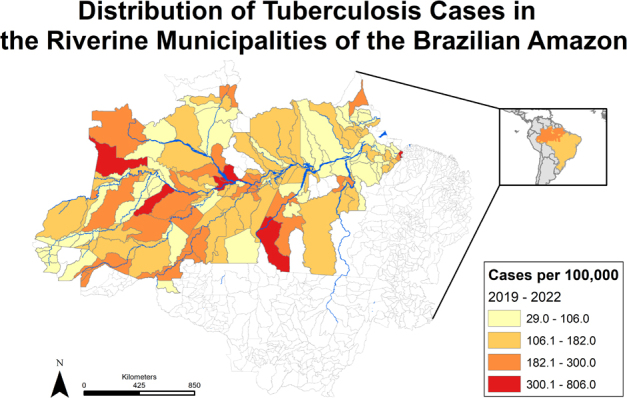
Incidence of tuberculosis in the riverine municipalities, aggregation from 2019 to 2022.

Figure 1 shows the distribution of cases per 100 thousand aggregated from the yearly incidence of TB in 2019–2022. Global spatial autocorrelation for this distribution, calculated through the Global Moran’s I method, yielded a value of 0.11. Monte Carlo simulations resulted in a p-value of 0.03, indicating statistically significant evidence of global spatial autocorrelation in this area. This result implies that the observed spatial distribution of TB incidence across municipalities is unlikely to have occurred by chance alone, and that municipalities with higher incidence are more likely to be neighbors to other municipalities with high incidence, the same being true for those municipalities with lower incidence.

**Figure 2:**
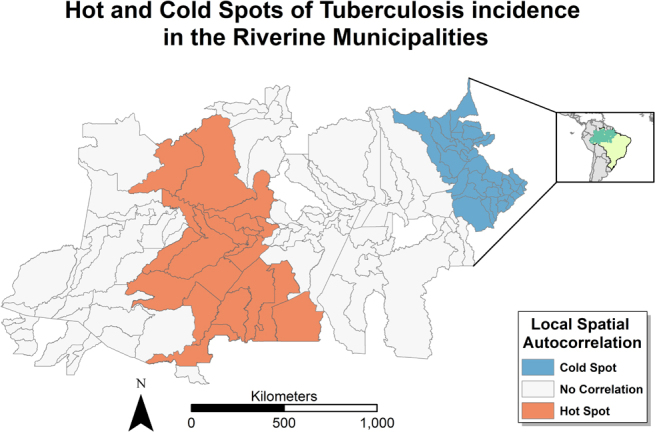
Local spatial autocorrelation with optimized Getis-Ord Gi*.

Figure 2 is a mapping representation of the Getis-Ord GI* analysis. The local indicators of spatial association, in our case the Optimized Getis-Ord GI*, identifies the exact areas where clusters occur. It indicates a clear west-to-east distinction in the geographical distribution of TB incidence in the last four years. Municipalities in the western portion of the studied area present with a cluster of high incidence and a cluster of low incidence can be found to the East, closer to the Atlantic Ocean.

**Figure 3:**
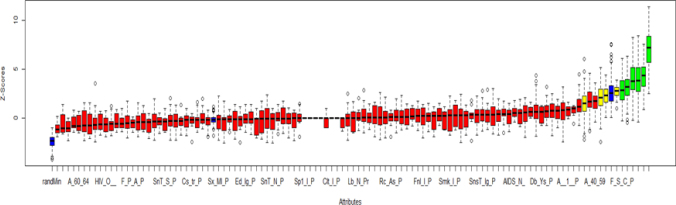
Variable selection using the Boruta method. Randomly generated (noise) variables are shown as blue boxes. Red are those rejected by the algorithm, green for acceptance, and yellow for those not classified by the algorithm. Variable names are shown in supplementary material 1.

Figure 3 displays the distribution of z-scores across the iterations of the Boruta Method for each variable. Only original surveillance variables with a median z-score for accuracy better than the best shuffled variable were used to train the final ML model.

Six surveillance variables were selected by the Boruta method for the analysis of hot spots vs. non-hot spots. These were: cases reported as new, cases reported as recurrent, cases reported as recurrent after abandonment, final outcome reported as death due to TB, final outcome reported as antibiotic treatment alteration and percentage of patients classified as smokers.

**Figure 4:**
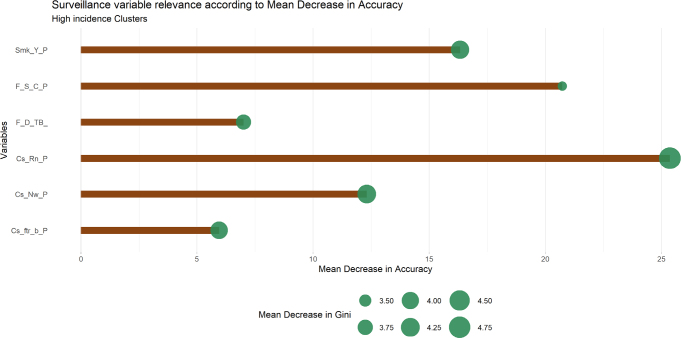
Variable importance in the hot spot prediction model based on mean decrease accuracy and Gini. From top to bottom, municipality variables are: percentage of cases with smoking history (Smk_Y_P), percentage of cases in which final outcome was antibiotic regimen change (F_S_C_P), percentage of cases in which final outcome was death due to TB (F_D_TB_), percentage of cases reported as recurrent cases (Cs_Rn_P), percentage of cases reported as new cases (Cs_Nw_P), percentage of cases reported as recurrent infection after abandoning treatment (Cs_ftr_b_P).

The values of variable importance can be summarized in Figure 4. Each surveillance variable’s importance can be quantified by the decrease in model accuracy if removed (horizontal axis value) and a decrease in Gini if removed (size of points).

**Figure 5:**
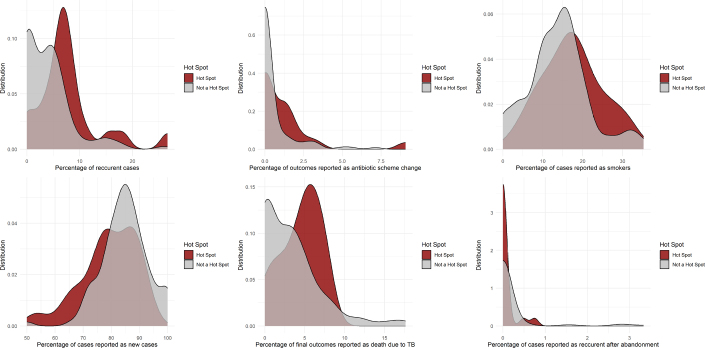
Density plots of most relevant predictor variables by status as hot spot.

For those selected surveillance features, the distribution of the municipality’s percentages is represented in Figure 5. Notably, a higher percentage of recurrent cases, cases involving antibiotic scheme changes, patients with a smoking history, and TB-related deaths are seen in hot spot municipalities. Conversely, newly reported cases tend to be less frequent in hot spots compared to non-hot spots.

**Figure 6:**
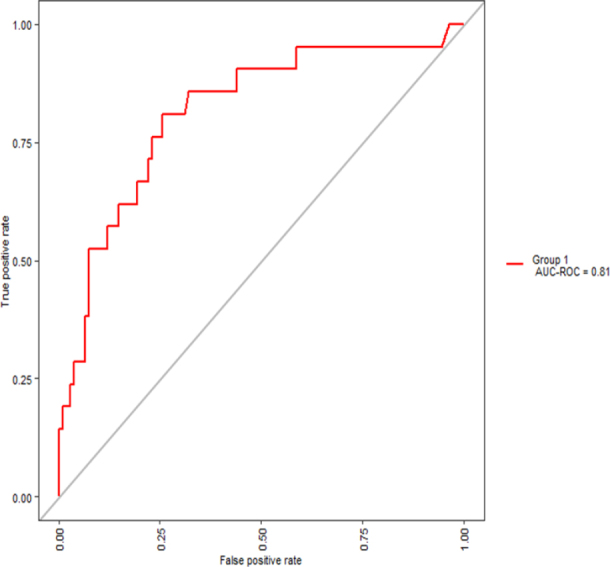
Receiver operator curve demonstrating the performance of the hot spot prediction model.

After adjusting for the best prediction cut-off point based on informedness, the relevant model performance metrics can be seen in Table 1. The model is 81% sensitive to predict high incidence municipalities of TB in the studied area, with a specificity of 74%. The Area Under the Receiver Operator Curve (AUC-ROC) is 0.81, with Figure 6 displaying the Receiver Operator Curve.

**Table 1:** Cross-validation of random forest classification predictor for high incidence clusters (k=10).

Performance metric	Value
Sensitivity	0.81
Specificity	0.74
Area under the curve – Receiver Operator Curve	0.81

## DISCUSSION

As an understudied and underserved group, the riverine population of the Amazon lacks evidence-based approaches to disease control. Most studies related to this population focus on specific rivers or sub-areas in the Amazon. Broader studies are thus important for a more general comprehension of the distribution of social determinants of health and diseases in the region. By providing a comprehensive definition of what constitutes a riverine municipality, our study provides a geospatial basis for this study of incident TB and for future studies of disease and health.

Using spatial autocorrelation analysis, the study identified evidence of clusters within the riverine municipalities of the Amazon. Notably, distinct high and low-incidence clusters were observed, demonstrating a clear demarcation from west (high-incidence) to east (low-incidence).

It is believed that one of the reasons for the disparity between riverine municipalities might be due to the costs related to transport of resources to each municipality. Since these municipalities rely heavily on rivers as their main mode of transportation, being further away from the Atlantic Ocean could result in higher operational costs to allocate healthcare resources, which are usually sourced from other regions of Brazil or imported from other countries. As such, riverine municipalities furthest away from the Atlantic Ocean might suffer from a lack of resources in the control of TB wen compared to those in the coastal region.

Due to its inherent capability to perform prediction tasks, ML techniques have been increasingly utilized in the study of diseases in populations, being successfully employed in public health research, with notable examples being found in the study of air pollution^23^, arboviruses^24^, COVID-19^25^ and TB^26^. Similarly, we employed ML models to better understand the most influential TB surveillance variables in the incidence of disease in the region.

Variable selection through the Boruta method revealed six specific surveillance variables as key for hot spot prediction (cases reported as new, cases reported as recurrent, cases reported as recurrent after abandonment, final outcome reported as death due to TB, final outcome reported as antibiotic treatment alteration and patients classified as smokers). Out of all comorbidities considered in this analysis, only smoking seems to be a relevant predictor of high incidence municipalities.

The predictive power of our model, exemplified by a cross-validated AUC-ROC exceeding 0.8, attests to its robustness, underscoring its potential applicability for public health advocates and policy makers.

Furthermore, through data visualization analysis of the most pertinent variables and their data distribution, we can better understand how the model predicts hot spots of disease. The density plot in Figure 5 reinforces that municipalities with a higher percentage of recurrent cases are more likely to be hot spots for the disease. It also demonstrates that places classified as hot spots tend to have a higher percentage of cases in which outcomes were classified as deaths due to TB, and as having antibiotic regimen change. Finally, it reveals that in disease hot spots, the proportion of cases involving smokers typically surpasses 15%.

Limitations of our approach include the fact that the prediction was performed in a cross-sectional manner, providing only a snapshot in time of which surveillance variables are correlated with TB hot spots. Future work should address predictions forward in time by considering whether surveillance variables in the SINAN system could predict future TB incidence distributions.

It is relevant to highlight that both the World Health Organization and the Brazilian Health Ministry recognize that the COVID-19 pandemic impacted the number of disease notifications for non-COVID diseases^1,2^. Starting from 2020, both entities acknowledge that a relative decrease in the total number of cases is primarily due to social distancing measures limiting access to healthcare and does not reflect an actual drop in total cases. More specifically for Brazil, the yearly incidence was in a consistent upward trend from 2016 to 2019 and subsequently presented a relative decrease in 2020 and 2021. These might have influenced the current analysis, and underreporting of cases should be considered upon generalization of these findings.

Our findings hold significant implications for public health authorities, offering a valuable data-driven tool to locate TB incidence clusters and determine their main associated surveillance variables. By identifying the geographical distribution of hot spots of disease incidence and developing an ML model that can predict them, we hope to fill the current gap in knowledge related to the study of TB in the Amazon and aid national and local authorities with an evidenced-based tool to direct resource allocation for disease control in the riverine municipalities.
