# Supplementary material for: Prediction of tuberculosis clusters in the riverine municipalities of the Brazilian Amazon with machine learning
Source: Rev Bras Epidemiol. 2024 May 13;27:e240024. doi: 10.1590/1980-549720240024 (PMC11093519; doi:10.1590/1980-549720240024)
Supplement: Supplementary file 1 [file 1980-5497-rbepid-27-e240024-Material-suplementar-1.pdf]

Variable	Meaning
Sp1_I_P	Percentage of TB cases in which Initial Sputum Sample was not reported
Sp1_P_P	Percentage of TB cases in which Initial Sputum Sample was reported as Positive
Spt1_Ng_P	Percentage of TB cases in which Initial Sputum Sample was reported as Negative
Spt1_Nt_P	Percentage of TB cases in which Initial Sputum Sample was reported as not being collected
S1_DNA_	Percentage of TB cases in which Initial Sputum Sample was deemed not applicable for collection, mainly involving extra-pulmonary TB cases.
AIDS_I_	Percentage of TB cases in which AIDS status was not reported
AIDS_Y_	Percentage of TB cases in which AIDS status was reported as Positive
AIDS_N_	Percentage of TB cases in which AIDS status was reported as Negative
Alc_I_P	Percentage of TB cases in which Alcoholism status was Not Reported
Alc_Y_P	Percentage of TB cases in which Alcoholism status was reported as Positive
Alc_N_P	Percentage of TB cases in which Alcoholism status was reported as Negative
Spt2m_I_P	Percentage of TB cases in which 2nd Month Sputum Sample was not reported
Spt2m_P_P	Percentage of TB cases in which 2nd Month Sputum Sample was reported as Positive
Spt2m_Ng_P	Percentage of TB cases in which 2nd Month Sputum Sample was reported as Negative
Spt2m_Nt_P	Percentage of TB cases in which 2nd Month Sputum Sample was reported as not being collected
S2_DNA_	Percentage of TB cases in which 2nd Month Sputum Sample was deemed not applicable for collection, mainly involving extra-pulmonary TB cases.
Sp6_I_P	Percentage of TB cases in which 6th Month Sputum Sample was not reported
Sp6_P_P	Percentage of TB cases in which 6th Month Sputum Sample was reported as positive
Spt6m_Ng_P	Percentage of TB cases in which 6th Month Sputum Sample was reported as Negative
Spt6m_Nt_P	Percentage of TB cases in which 6th Month Sputum Sample was not reported
S6_DNA_	Percentage of TB cases in which 6th Month Sputum Sample was deemed not applicable for collection, mainly involving extra-pulmonary TB cases.
Bn_Ig_P	Percentage of TB cases in which Welfare Recipient Status was not reported
Bn_Ys_P	Percentage of TB cases in which Welfare Recipient Status was reported as Positive
Bn_N_Pr	Percentage of TB cases in which Welfare Recipient Status was reported as Negative
Ag_I__P	Percentage of TB cases in which Age was not reported
A__1__P	Percentage of TB cases reported as younger than 1 year of age
A_1_4__	Percentage of TB cases reported between 1 and 4 years of age
A_5_9__	Percentage of TB cases reported between 5 and 9 years of age
A_10_14	Percentage of TB cases reported between 10 and 14 years of age
A_15_19	Percentage of TB cases reported between 15 and 19 years of age
A_20_39	Percentage of TB cases reported between 20 and 39 years of age
A_40_59	Percentage of TB cases reported between 40 and 59 years of age
A_60_64	Percentage of TB cases reported between 60 and 64 years of age
A_65_69	Percentage of TB cases reported between 65 and 69 years of age
A_70_79	Percentage of TB cases reported between 70 and 79 years of age
A_80___	Percentage of TB cases reported above 80 years of age
Cs_Nw_P	Percentage of TB cases reported as New Infections
Cs_Rn_P	Percentage of TB cases reported as Recurrent Infections
Cs_ftr_b_P	Percentage of TB cases reported as Recurent Infection after abandoning treatment

Cs_nk_P	Percentage of TB cases reported as Not Know Previous Status
Cs_tr_P	Percentage of TB cases reported as Transferred from another Municipality
Cs_ftr_d_P	Percentage of TB cases reported as Infection Diagnosed after Death
Lb_Ys_P	Percentage of TB cases reported as having positive Laboratory Confirmation
Lb_N_Pr	Percentage of TB cases reported as having negative Laboratory Confirmation
Clt_I_P	Percentage of TB cases in which Culture Result was not reported
Clt_P_P	Percentage of TB cases in which Culture Result was Reported as Positive for TB
Clt_Ng_P	Percentage of TB cases in which Culture Result was Reported as Negative for TB
Clt_n_P	Percentage of TB cases in which Culture Result was Reported as On Going
Clt_Nt_P	Percentage of TB cases in which Culture Result was Not Reported
Db_Ig_P	Percentage of TB cases in which Diabetes status was not reported
Db_Ys_P	Percentage of TB cases in which Diabetes status was Reported as Positive
Db_N_Pr	Percentage of TB cases in which Diabetes status was Reported as Negative
Ed_Ig_P	Percentage of TB cases in which Education Level was not reported
Ed_ll_P	Percentage of TB cases reported as being Illiterate
Ed_nc_l_P	Percentage of TB cases reported as having started but not completed Elementary School
Ed_cm_l_P	Percentage of TB cases reported as having completed Elementary School
Ed_nc_m_P	Percentage of TB cases reported as having started but not completed Middle School
Ed_cm_m_P	Percentage of TB cases reported as having completed Middle School
Ed_nc_h_P	Percentage of TB cases reported as having started but not completed High School
Ed_cm_h_P	Percentage of TB cases reported as having not completed High School
Ed_nc_c_P	Percentage of TB cases reported as having started but not completed College
Ed_cm_c_P	Percentage of TB cases reported as having completed College
E_DNA_P	Percentage of TB in which education was deemed not applicable for collection, mostly children before age of enrollment in Elementary School
Frm_I_P	Percentage of TB cases in which Form of Disease was not Reported
Frm_P_P	Percentage of TB cases in which Form of Disease was Reported as Pulmonary
F_E_P_P	Percentage of TB cases in which Form of Disease was Reported as Extra-Pulmonary
Frm_c_P	Percentage of TB cases in which Form of Disease was Reported as Pulmonary + Extra-Pulmonary
HIV_I_P	Percentage of TB cases in which HIV Initial Treatment Testing Status was Not Reported
HIV_P_P	Percentage of TB cases in which HIV Initial Treatment Testing Status was reported as Positive
HIV_Ng_P	Percentage of TB cases in which HIV Initial Treatment Testing Status was reported as Negative
HIV_O__	Percentage of TB cases in which HIV Initial Treatment Testing Status was reported as "Testing On Going"
HIV_Nt_P	Percentage of TB cases in which HIV Initial Treatment Testing Status was reported as not collected
Rc_Ig_P	Percentage of TB cases in which Race was Not Reported
Rc_Wh_P	Percentage of TB cases in which Race was reported as White
Rc_Bl_P	Percentage of TB cases in which Race was reported as Black
Rc_As_P	Percentage of TB cases in which Race was reported as Asian
Rc_Mx_P	Percentage of TB cases in which Race was reported as Mixed Race
Rc_Nt_P	Percentage of TB cases in which Race was reported as Native
Sx_Ig_P	Percentage of TB cases in which Sex was Not Reported
Sx_Ml_P	Percentage of TB cases in which Sex was Reproted as Male

Sx_Fm_P	Percentage of TB cases in which Sex was Reported as Female
Fnl_I_P	Percentage of TB cases in which Final outcome as not collected
Fnl_C_P	Percentage of TB cases in which Final outcome was reported as cure
Fnl_A_P	Percentage of TB cases in which Final outcome was reported as Abandoning Treatment
F_D_TB_	Percentage of TB cases in which Final outcome was reported as Death by TB
F_D_N_P	Percentage of TB cases in which Final outcome was reported as Death not by TB
Fnl_T_P	Percentage of TB cases in which Final outcome was reported as Transferred to Another Municipality
F_DR_TB	Percentage of TB cases in which Final outcome was reported as Multi-Drug Resistant TB
F_S_C_P	Percentage of TB cases in which Final outcome was reported as Antibiotic Regimen Change
F_T_F_P	Percentage of TB cases in which Final outcome was reported as Treatment Failure
F_P_A_P	Percentage of TB cases in which Final outcome was reported as Primary Abandonment
Smk_I_P	Percentage of TB cases in which Smoking status was not reported
Smk_Y_P	Percentage of TB cases in which Smoking status was reported as Positive
Smk_N_P	Percentage of TB cases in which Smoking status was reported as Negative
OT_Ig_P	Percentage of TB cases in which "directly observed treatment" status was not reported
OT_Ys_P	Percentage of TB cases in which "directly observed treatment" status was reported as Positive
OT_N_Pr	Percentage of TB cases in which "directly observed treatment" status was reported as Negative
SnsT_Ig_P	Percentage of TB cases in which Sensitivity Test Status (GeneXepert) was not reported
SnsT_rI_P	Percentage of TB cases in which Sensitivity Test Status (GeneXepert) was reported as Resistant to Isoniazid
SnT_R_P	Percentage of TB cases in which Sensitivity Test Status (GeneXepert) was reported as Resistant to Rifampin
ST_IR_P	Percentage of TB cases in which Sensitivity Test Status (GeneXepert) was reported as Resistant to Isoniazid and Rifampin
ST_MDR_	Percentage of TB cases in which Sensitivity Test Status (GeneXepert) was reported as Multi-Drug Resistant
SnT_S_P	Percentage of TB cases in which Sensitivity Test Status (GeneXepert) was reported as Sensitive to First-Line Treatment Antiobiotics
ST_O__P	Percentage of TB cases in which Sensitivity Test Status (GeneXepert) was reported as On Going
SnT_N_P	Percentage of TB cases in which Sensitivity Test Status (GeneXepert) was reported as Not Collected
