# Supplementary figures and images for: Prediction of tuberculosis clusters in the riverine municipalities of the Brazilian Amazon with machine learning
Source: Rev Bras Epidemiol. 2024 May 13;27:e240024. doi: 10.1590/1980-549720240024 (PMC11093519; doi:10.1590/1980-549720240024)

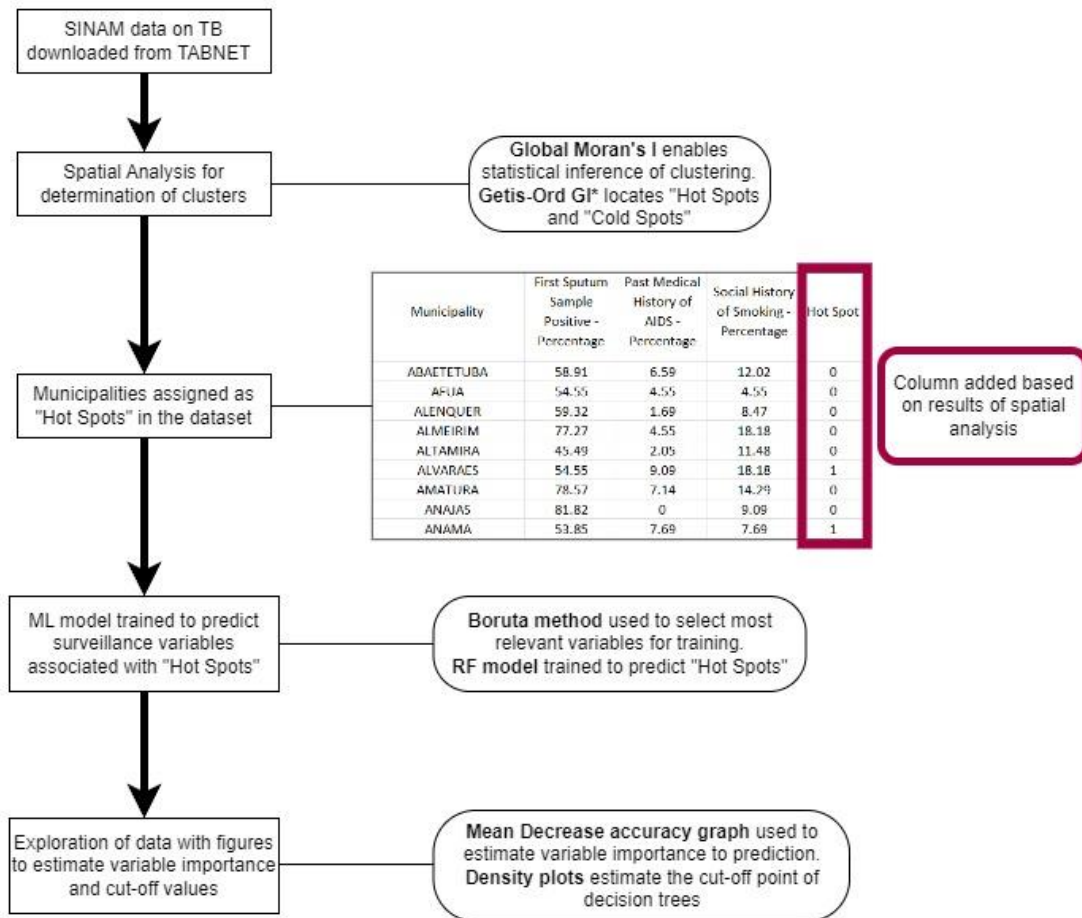

Complementary material 3 – Flowchart of the data analysis pipeline of this study.

Supplement: Supplementary file 2 [file 1980-5497-rbepid-27-e240024-Material-suplementar-2.pdf]
